# P-12. Evaluating the Epidemiological Impact of Lowering the Recommended Age for Adult Pneumococcal Vaccination in the United States

**DOI:** 10.1093/ofid/ofae631.222

**Published:** 2025-01-29

**Authors:** Kevin Bakker, Giulio Meleleo, Oluwaseun Sharomi, Robert Nachbar, Rachel Oidtman

**Affiliations:** Merck & Co., Inc., Rahway, NJ, USA, Philadelphia, Pennsylvania; Wolfram Research, Inc., Champaign, Illinois; Merck & Co., Inc., Lansdale, Pennsylvania; Wolfram Research, Inc., Champaign, Illinois; Merck & Co., Inc., Lansdale, Pennsylvania

## Abstract

**Background:**

In the United States, there are two age-based pneumococcal vaccine recommendations – children aged 0-2 years and adults aged 65+ years. Adults aged 50-64 are recommended to receive a pneumococcal vaccine if at increased risk of pneumococcal disease; disease burden in this age groups represents the third highest level of pneumococcal disease. PCV20, a 20-valent PCV, and PCV15 are higher valent vaccines currently approved for adults. V116, an investigational adult specific PCV is being developed to specifically broaden protection in the adult population, by including key serotypes in currently licensed vaccines and 8 unique serotypes responsible for ∼30% of pneumococcal disease in adults in the US.

**Figure:**
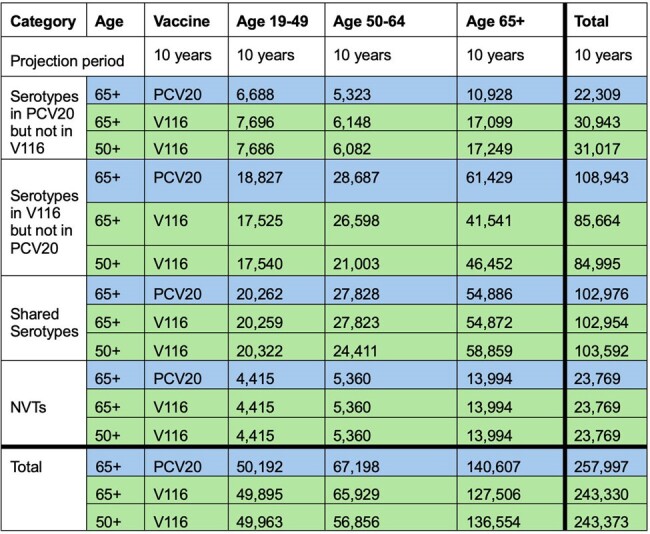

Cumulative IPD cases in a 10-year projection period for V116 and PCV20.

**Methods:**

We evaluated the impact of lowering the pneumococcal adult age-based vaccine recommendations in the United States to individuals aged 50+ years using a model that tracked age- and serotype-specific *Streptococcus pneumoniae* (SP) transmission. The model was calibrated to historical age- and serotype-specific invasive pneumococcal disease (IPD) data in the United States spanning 2000-2019. We evaluated the impact of lowering the recommended vaccination age in adults to 50+ years-old with V116. For projections we maintained an 80/20 coverage of PCV20/PCV15 in 82% of children and assumed a VCR of 57% for adults aged 65+. In the 50+ years old scenario, we reduced VCR to 46%, which was the age-specific tetanus rates for individuals aged 50-64 years-old multiplied by the reduction in uptake from the Shingrix vaccine when the recommended age was lowered. These adult VCR values assume a PCV was received in the last 10 years.

**Results:**

Reducing the adult recommended vaccination age to 50+ years with the reduced VCR, led to similar overall reductions in IPD incidence when compared to maintaining the vaccination age at 65+ years (Table 1). The additional reductions in IPD in 50-64 year-olds was negated by the increase in cases in individuals aged 65+ years, due to vaccine waning.

**Conclusion:**

If the recommended pneumococcal vaccination age for adults is reduced, additional efforts must be made to minimize the reduction of VCR, otherwise total IPD incidence will be similar to the current 65+ year-old age recommendation due to vaccine waning.

**Disclosures:**

**Kevin Bakker, PhD**, Merck & Co., Inc.: Grant/Research Support|Merck & Co., Inc.: Stocks/Bonds (Public Company) **Giulio Meleleo, PhD**, Merck & Co., Inc.: Vendor **Oluwaseun Sharomi, MSc, PhD**, Merck & Co., Inc.: Full time employee|Merck & Co., Inc.: Stocks/Bonds (Public Company) **Robert Nachbar, PhD**, Merck & Co., Inc.: US 7,219,020, US 5,292,741|Merck & Co., Inc.: Vendor|Merck & Co., Inc.: Stocks/Bonds (Public Company) **Rachel Oidtman, PhD**, Merck & Co., Inc.: Full time employee|Merck & Co., Inc.: Stocks/Bonds (Public Company)

